# Seed Endophytic *Achromobacter* sp. F23KW as a Promising Growth Promoter and Biocontrol of Rhizoctonia Root Rot of Fenugreek

**DOI:** 10.3390/molecules27175546

**Published:** 2022-08-29

**Authors:** Ehsan M. Rashad, Dalia M. Shaheen, Abdulaziz A. Al-Askar, Khalid M. Ghoneem, Amr Abker Arishi, El Sayed A. Hassan, WesamEldin I. A. Saber

**Affiliations:** 1 Seed Pathology Research Department, Plant Pathology Research Institute, Agricultural Research Center, Giza 12619, Egypt; 2 Botany and Microbiology Department, Faculty of Science, King Saud University, Riyadh 11451, Saudi Arabia; 3 School of Molecular Sciences, The University of Western Australia, Perth, WA 6009, Australia; 4 Seed Technology Research Department, Field Crops Research Institute, Agricultural Research Center, Giza 12619, Egypt; 5 Microbial Activity Unit, Microbiology Department, Soils, Water and Environment Research Institute, Agricultural Research Center, Giza 12619, Egypt

**Keywords:** bacteria, seed endophytes, *Rhizoctonia solani*, natural products, lytic enzymes, plant growth, seed germination, indole acetic acid

## Abstract

Root rot is one of the most significant soil and seed-borne fungal diseases, limiting the cultivation of fenugreek plants. Endophytic bacteria and their natural bioproducts have emerged as growth promoters and disease suppressors in the current era. Despite limited research, seeds are a good funder of endophytic microbiomes, which are transmitted from them to other seedling parts, thereby providing a shield against biotic and abiotic anxiety and promoting the growth at early germination and later stages. The current study evaluated the hypothesis that seed endophytic bacteria and their lytic enzymes, growth promotors, and antifungal molecules can induce growth, and inhibit root rot disease development at the same time. The isolation trial from fenugreek seeds revealed a lytic *Achromobacter* sp., which produces indole acetic acid, has antifungal compounds (e.g., 2-Butanol, 3,3’-oxybis-), and reduces the growth of *Rhizoctonia solani* by 43.75%. Under the greenhouse and natural field conditions, bacterial cells and/or supernatant improved the growth, physiology, and yield performance of fenugreek plants, and effectively suppressed the progress of root rot disease; this is the first extensive study that uses a new seed-borne endophytic bacterium as a plant-growth-promoting, and biocontrol tool against the sclerotia-forming; *R. solani*; the causative of fenugreek root rot.

## 1. Introduction

Fungi are widespread, cause severe diseases, and secrete mycotoxins under wide environmental conditions; consequently, exceptional consideration has been rewarded for diseases caused by pathogenic fungi due to the direct destructive impact. Yet, fungal infections are not limited to a certain group of living organisms, they can also infect a wide array of creatures.

The annual *Trigonella foenum-graecum* L. (fenugreek) belongs to the *Papilionaceae* subfamily of the *Fabaceae* family. Fenugreek plant is native to western Asia, Northern Africa, and southeastern European countries [1]; however, its good adaptability to different climatic and environmental conditions makes it widespread and appropriate for planting in different habitats, including Asia, Africa, Europe, America, and Australia [2]. India, Ethiopia, Egypt, and Turkey are the major seed producers [3]. Seeds are considered an important food ingredient, flavoring agent, and spice, nutritionally, fenugreek seeds contain 58% carbohydrates, 23% protein, 6% fat, and 9% water, in addition to vitamins, and minerals especially manganese, calcium, and iron [2]. As a medicinal plant, the seeds contain many active compounds, of which five classes of constituents (saponins, flavonoids, alkaloids, coumarins, and vitamins) have medicinal and pharmaceutical applications, and are mainly attributed to the biological and therapeutic activities [4], such as antiaging, antidiabetic, anticancer, antimicrobial and cardioprotective [1,2,4].

Unfortunately, substantial economic losses occur due to attacking fenugreek plants by various fungal pathogens, such as *Cercospora traversiana* (leaf spot), *Erysiphe polygoni* and *Leveillula taurica* (powdery mildew), *Peronospora trigonellae* (downy mildew), *Uromyces anthyllidis* (rust), *Fusarium oxysporum* (wilt), and *Macrophomina phaseolina* (charcoal rot) [5].

*Rhizoctonia solani* Kühn (teleomorph: *Thanatephorus cucumeris* (A. B. Frank) Donk., order *Ceratobasidiales* of *Basidiomycota*), is a ubiquitous soil-borne phytopathogen, with a broad host range and strains [5,6]. Sclerotia formed by *Rhizoctonia* strains have thick outer layers (1–3 mm in diameter), containing food stocks, that enable them to persist in the soil for a long time [7]. Under appropriate conditions, the germinated sclerotia enter the host plant through a straight invasion of the cuticle/epidermis layer or through ordinary vents, which is often accompanied by the secretion of several hydrolytic enzymes, leading to the development of several severe diseases such as collar and, root rots, wire stem, and damping off [5,6,8]. The pathogen was also reported to cause foot rot and reddish-brown cankers on root and stem near the ground level of fenugreek plants causing heavy casualties under appropriate conditions [5].

Under field conditions, primarily, *R. solani* severely attacks fenugreek seeds before or after germination, causing pre-emergence damping-off, while in the early stages of germination, causing post-emergence damping-off [6,9]; it is also capable of infecting the other green parts, and the hypocotyls [5]. The infected roots are poorly developed, finer roots are either rotted or not formed, leading to stunting growth and the plant can easily be pulled out [8]. *Rhizoctonia solani* spreads vigorously under natural field conditions, causing about 55% yield loss in fenugreek yield; therefore, *R. solani* is considered the most destructive, and main constraint for profitable production worldwide [5,6]; it is not expected to full eradication the pathogen, but its severity can be curtailed through mainly chemical fungicides, which are questioned owing to the lethal impact on beneficial organisms.

Innovatively, endophytic microorganisms are a major category of plant symbionts that dwell within robust plant tissues without developing diseases, which are connected with the plant during its lifetime, from seed germination to the fruiting process; however, microbes of seed-borne endophytes have not been, totally, discovered yet; this type of endophytes is of pronounced concern owing to the potential to produce natural phytohormones, lytic enzymes, and other secondary natural products, as well as enhance soil structure, and bioremediate contaminated soils that improve plant biomass and yield under abiotic and biotic stresses, importantly, seed-borne endophytes can effectively control or hinder phytopathogens. Although few studies, seed-borne endophytic microbes showed high bioactivity and biodiversity in their communities [10,11].

Because of the complex components, the plant tissue structure restricts the movement and activity of the endophytic microorganisms inside plant tissues. Therefore, the important role of many hydrolytic enzymes produced by endophytes becomes clear. Plant tissue is created essentially from hemicellulose, cellulose, pectin, and protein. The enzymes that degrade such components are regarded as cell wall-degrading enzymes that ease the access of the microbes through plant tissue and may be engaged in the biocontrol process of other pathogens [12,13]. Enzymes are recognized as biocatalysts that perform an abundance of chemical reactions, the enzymatic profile of microorganisms differed greatly according to the ecological conditions, the genetic structure, and the host plant [10]. The overall cellulolytic activity is performed mainly through cellulase-releasing monomers of glucose units [10]. Whereas, whereas xylanase manages the hydrolysis of hemicellulose, releasing xylose [14,15]. The pectin polymer that presents in plants cell is catalyzed by the hydrolysis of the pectinase [12,16]. Proteases are in charge of cleaving the protein into peptides and amino acids [13]. The chitinolytic activity was discovered in limited numbers of the tested bacteria. Chitinases catalyze the hydrolysis of pathogenic fungi, therefore, chitinase is considered a biological control agent against fungi and insects [17,18]. Endophytes may also have some activity in the production of natural plant growth promotors, e.g., indole acetic acid (IAA), which has a chief task in leaf morphogenesis and vasculature system differentiation [19].

Limited knowledge is available about the bacterial endophytes naturally occurring in fenugreek seeds. Our study has a head start in investigating this point. Herein, a novel endophytic bacterial isolate (*Achromobacter* sp. strain F23KW) was reported from the fenugreek seeds. The bacterium was screened for the profile of lytic enzymes, IAA, and antifungal compounds as a biological system capable of inducing growth and control of the fungal pathogen. The in vitro and in vivo capabilities of the biologically active bacterium to control *R. solani* were evaluated. The bacterial strain was appraised as an inhibitor to the growth of root rot pathogen and for growth promotion of fenugreek plants as well.

## 2. Materials and Methods

### 2.1. The Pathogenic Fungus

A highly pathogenic *Rhizoctonia solani* RT8 was delivered by Seed Pathology Research Department, Plant Pathology Research Institute, Agricultural Research Center, Giza, Egypt. The pathogen was subcultured on potato dextrose agar (PDA) plates (Difco, Georgia, GA, USA), and incubated (25 ± 2 °C) for 5 days.

### 2.2. Preparation of R. solani Inoculum

Inoculum of *R. solani* was formulated by growing plates of PDA (25 ± 2 °C), after five days, the plugs of mycelium were used to inoculate sterilized sorghum: coarse sand: water (2:1:2 *v*/*v*) medium in 500 mL Erlenmeyer flasks and incubated at 25 °C for 15 days. 

### 2.3. Isolation of Endophytic Bacterial Microbiome

Twenty samples of healthy fenugreek seeds were gathered from various Egyptian governorates (Qena, Assuit, Sohag, Aswan, Al-Menia, Al-Fayoum, Beni Suef, Al-Sharkia, Al-Ismailia, and Kafr El-Sheikh) lie within 22°29′ N and 31°31′ N (latitudes), and 29°26′ E and 33°03′ E (longitudes), during February and March 2022. The samples were collected from an area of 50 m × 50 m in a random zigzag design. The matured fenugreek pods were collected from fields and labeled, then preserved at 4 °C. The endophytic seed-borne bacteria were isolated, utilizing the agar plate procedure [20]. The seed sample (200 healthy-looking seeds) was surface sterilized with ethanol (70% for one min), then sodium hypochlorite (2% for 90 s), followed by ethanol (100% for 30 s); finally, the seeds were rinsed with sterilized distilled water over a 10 min and dried using sterilized filter paper under laminar airflow chamber. About 10 seeds were plated on a nutrient agar plate (Nutrient Agar, BD, Difico™ Georgia, GA, USA), then incubated at 25 ± 2 °C below cool white fluorescent light for 12 h light/darkness cycle for 72 h. Colonies that appeared around each seed were picked after 48 h. The bacterial pure culture was obtained by spreading them on the surface of nutrient agar plates to obtain a single colony. The isolates were maintained and regularly subcultured on nutrient agar slants.

### 2.4. In Vitro Evaluation of Endophytic Bacteria against R. solani

A total of 28 bacterial endophytes isolated from samples of fenugreek seeds collected from different locations were assessed against *R. solani* using the dual culture plate procedure [11]. A disc (5 mm), acquired from 5-day culture of *R. solani*, was placed 1 cm apart from the border of each PDA plate, and a loop of each bacterial isolate was streaked 1 cm apart from the opposite edge of the plate. Plates with pathogen alone served as control. The plates were incubated at 25 °C. The test ended when fungal growth was wholly spread over the control plates. The following formula was used to estimate the inhibition in growth:

$$\mathrm{Fungal}\;\mathrm{growth}\;\mathrm{inhibition}=\frac{R1-R2}{R1}\times 100$$

where R1 is the linear inward growth of the pathogen in the control plate and R2 = the linear inward growth of the pathogen in the dual culture plate.

### 2.5. Biochemical Profile of Endophytic Bacteria

#### 2.5.1. Bacterial Inoculum

One mL of bacterial inoculum (prepared by scrapping bacterial slants using 5 mL sterilized tap water) was used to inject 100 mL sterilized nutrient broth medium in 500-mL Erlenmeyer flasks containing. The initial pH was adjusted to 7 and then sterilized at 121 °C for 15 min. The inoculated medium was incubated at 30 °C under shaking (150 rpm) for 2 days, followed by centrifugation for 15 min at 4 °C at 5000 rpm. The bacterial cells were washed and re-suspended in sterile distilled water; then, the viable bacterial count was determined by serial dilutions using standard plate techniques, and finally, the count was adjusted to 10^8^ cfu/mL. The bacterial supernatant was kept under cooling till used.

#### 2.5.2. Culturing Technique

For screening the isolated endophytic bacteria for lytic activity, and IAA secretion. The procedure of solid-state fermentation (SSF) was utilized, applying one gram of dried and ground fenugreek plants as a substrate in 250-mL Erlenmeyer flasks. The dried residual was moistened with tap water (5 mL, pH 7.2), and autoclaved (121 °C for 15 min), then inoculated with 1.0 mL of previously prepared inoculum. The batch fermentation was incubated in the dark for 7-days at 30 °C. 10 mL of 0.01% Tween 80 was mixed with the grown culture and shaken for 30 min on a rotary shaker (150 rpm). The bacterial supernatant was then separated through filtration, followed by centrifugation at 5000 rpm for 20 min. The resulting post-culture supernatant was examined for hydrolytic enzymes, indole acetic acid content, and pH.

#### 2.5.3. Assay of Hydrolytic Enzymes 

Xylanase and filter paperase (FPase) were assayed in the cultural supernatant using xylan [14], and microcrystalline cellulose [12] as substrates, respectively. Each substrate (0.5%) was mixed in 0.05 M citrate buffer (pH 4.8). Equal amounts of the supernatant and substrate-buffer solution were mixed and incubated (50 °C) for 30, and 60 min, respectively. Then, the reducing units were determined.

Polygalacturonase (PGase) was measured in a reaction mixture composed of the bacterial supernatant and 0.1 M sodium acetate buffer (pH 5.2), containing pectin as a substrate. After 30 min incubation at 40 °C [21], then, the reducing units were determined.

The liberated reducing units by the three enzymatic actions were spectrophotometrically measured at A_575_ nm by the 3,5-dinitrosalicylic acid (DNSA) routine [22]. With the aid of standard curves, and under the assay situations, the unit (U) of the three enzymes was defined as the amount of enzyme required to release one µmol/g/min of reducing units of xylose for xylanase, glucose for FPase, or D-galacturonic acid monohydrate for PGase.

The proteolysis activity (protease) was assayed in a reaction mixture of casein as a substrate and the supernatant. The mixture was incubated (37 °C for 10 min), and the free amino acids were separated by trichloroacetic acid and measured at A_280_. One protease U, under the assay situations, was expressed as the amount of the protease that resulted in the liberation of one µg of tyrosine equivalent/g/min [10].

Chitinase was assayed, incubating reaction mixture of chitin azure (3-mg/mL), potassium phosphate buffer (0.2 M, pH 7), and supernatant at 30 °C for 30 min. The mixture was boiled for 5 min, then centrifuged at 13,000× *g* for 5 min. The absorbance was measured spectrophotometrically at A_575_ nm. Under the assay situations, one chitinase U was defined as the enzyme amount that cause an increase of 0.01 in the absorbance [17].

Additionally, the IAA secretion was measured in the bacterial supernatant [23]. The final supernatant pH was also measured.

### 2.6. Gas Chromatography-Mass Spectrometry (GC-MS)

The supernatant of the selected bacterium was analyzed using gas chromatography; 2010 Shimadzu capillary attached to the mass spectrometer unit (GC-MS–model QP 2010 (Shimadzu, Kyoto, Japan)); DB–5 ms nonpolar fused silica capillary column (30 m × 0.25 mm, 0.25 μm film thickness). The oven temperature system was 70 °C, 3 °C/min gradient to 200 °C for 35 min. Injection temperature at 200 °C with helium as the carrier gas at 1.0 mL/min, and linear velocity at 45.1 cm/s. The effluent of the GC column was presented into the supply of MS and spectra were acquired in the EI mode with ionization energy of 70 eV in the electronic ionization mode. The temperature of the ion source was 200 °C with 3 min of solvent cut time. The scanning of the mass analyzer was set between 40 and 1000 *m*/*z* at 240 °C. The sample constituents were identified by comparing the relative indices and mass spectra using libraries of WILEY and the National Institute of Standards and Technology.

### 2.7. Morphological, Biochemical, and Molecular Identification

Among the isolated endophytic bacterial isolates, the selected strain F23KW was investigated for morphological features. The colony was monitored after culturing on NA medium for 2 and 3 days at 27 ± 2 °C. The strain was tested for Gram reaction and motility. The strain was also investigated for the biochemical tests i.e., oxidase, catalase, urease, gelatin hydrolysis, phenylalanine, nitrate/nitrite reductase, lipid hydrolysis, indole, H_2_S production, carbohydrate fermentation, utilization of different carbon sources, production of enzymes, pH range of growth, and tolerance to NaCl were tested using standard protocols [24].

Molecular identification of bacterial isolate; F23KW was performed. The ribosomal RNA analysis of 16S was applied, in which the genomic DNA was isolated and the 16S rRNA gene was amplified using the forward (5′AGAGTTTGATCCTGGCTCAG 3′) and reverse (5′ GGTTACCTTGTTACGACTT 3′), primers. The acquired 16S rRNA gene sequence was aligned with the resembling 16S rRNA sequences of the characteristic category of bacterial strains retrieved from the databases of GenBank by using the BLAST program.

For evolutionary relationships, the Neighbor-Joining technique was applied to deduce the phylogenetic analysis [25]. The associated taxa were clustered together in the bootstrap test (1000 replicates) to estimate the percentage of replicate trees [26]. The ambiguous points were eliminated for each sequence pair by pairwise deletion option. The evolutionary study was conducted by MEGA11 [27].

### 2.8. Greenhouse Experiment 

The selected endophytic bacterium was assessed against *R. solani* under greenhouse situations. Fenugreek seeds (Giza 2 variety), kindly obtained from the Field Crops Research Institute, Agricultural Research Center, Giza, Egypt) susceptible to *Rhizoctonia* root rot infection were used in this study. Experimental pots containing 5 kg/pot disinfected soil (clay: sand at 2:1, *v*/*v*) were singly infested 0.4% (*w*/*w*) with the previous pathogen inoculum, then irrigated with tap water, and kept for one week to assure the dispersal of the fungus. Control pots were filled with steam-sterilized soil, and irrigated with water only. The surface of fenugreek seeds was sterilized utilizing sodium hypochlorite (1%), followed by washing with sterile water, and desiccated using filter paper. The surface sterilized seeds were immersed in the bacterial cells, bacterial supernatant (filtered through a 33 mm diameter sterile syringe filter with a 0.22 µm pore size), or their combination for 3 h, in the presence of Arabic gum to ensure the adhesion of bacterial cells on the seed surface and allowing them to air dry. Ten seeds were seeded per pot and fifteen replicates were used for each treatment.

The arrangement of the experimental treatments was divided into two main sections; infection treatments that involved; (1) *R. solani* pathogen only (P), (2) P plus endophytic bacterial cells (PBC), (3) P plus endophytic bacterial supernatant (PBS), (4) P plus a mixture of PBC and PBS (PBCS), and (5) P plus the recommended fungicide; Rhizolex-T 50% WP at 3 g/kg seed as adorning (PF). While the noninfected treatments were (6) the negative control without any treatment (control), (7) single application of endophytic bacterial cells (BC), (8) single application of endophytic bacterial supernatant (BS), and (9) a mixture of BC and BS (BCS). A randomized block design was used and all pots were kept in the greenhouse for 60 days at 28/22 °C, day/night temperature, and 12 h photoperiod.

#### 2.8.1. Disease Assessment

Disease development was determined through two measurements. After two weeks, the pre-emergence damping-off was determined as the percentage of seeds and seedling death before emergence (germination) in relation to the initial seeds number. Sixty days after planting, the post-emergence damping-off was determined as the percentage of seedling death after germination (post-infected seedlings) in relation to the initial seeds number, and survived plants were recorded as the percentage of living plants at the end of 60 days after planting.

#### 2.8.2. Growth Parameters

Eight weeks after sowing, the fenugreek growth parameters were measured. Ten plants were gently pulled out, washed, and assessed for the shoot and root lengths (cm), numbers of leaves, and fresh and dry weights (g) of the plant. The fresh weight of the shoot and root was measured after drying the plants for 24 h at room temperature, and the dry weight of plants was recorded after treatment in an oven at 70 °C for 2 days till constant weight.

#### 2.8.3. Estimation of Total Phenol

Total phenol was detected in fenugreek plants after 15 days from planting with the folin-ciocalteu reagent [28]. The fresh weight of shoot (0.5 g) was ground with a mortar and pestle in a 10-times volume of ethanol (80%), then centrifuged (20 min at 10,000 rpm). The extraction was repeated and the filtrates were collected together and dehydrated by evaporation. The deposit was liquified in 5 mL of distilled water. Aliquots of 0.2 mL were pipetted into a clean test tube and 2.8 mL of distilled water and 0.5 mL of folin reagent were added. Three minutes later, Na_2_CO_3_ (2 mL of 20%) was mixed thoroughly to each tube, then boiled for one min., and chilled. Then measured at A_650_ nm [29].

#### 2.8.4. Defense-Related Enzymes

Enzymes were extracted from leaves by homogenization for one minute in a mixture of Tris-HCl buffer (0.05 mM, pH 8.4), and 15 mM β-mercaptoethanol, followed by centrifugation (10,000 rpm) for 20 min at 4 °C [30]. The filtrate was completed to a definite volume with the same buffer.

The reaction mixture of polyphenol oxidase (PPO) contained 1 mL 0.2 M potassium phosphate buffer (pH 7), 0.5 mL filtrate, and 0.5 mL of catechol as a substrate. The variation in absorbance was assessed at A_420_ nm. The unit of the enzyme activity (U) was identified as ΔA_420_ min/g fresh weight under the assay conditions [31].

The assay mixture of peroxidase activity (POD) consisted of 0.5 mL of extract, 1 mL of phosphate buffer (pH 7.1), 0.5 mL pyrogallol, and 0.5 mM H_2_O_2_. The developed color, as a consequence of pyrogallol oxidation, was measured spectrophotometrically. The unit of the enzyme activity (U) was identified as ΔA_470_ min/g fresh weight under the assay conditions [31].

### 2.9. Field Trial

The field experiment was applied at Al-Qasasin farm, Agricultural Research Station, Agricultural Research Center, Ismailia, Egypt (30°47′64′′ N 31°66′58′′ E) in the 2022 winter season, to evaluate the endophytic bacterial treatments under natural growth conditions on the growth and yield and its components of fenugreek plants. The trial was performed under natural infection conditions. Five seed treatments were selected from the greenhouse trial to be reapplied in the open field environments. The treatments were; control, BC, BS, BCS, and the recommended fungicide (F). The cultivated area is sandy loamy soil. Physically, the soil is composed of 66.5% sand, 28.5% silt, 5% clay, and organic carbon of 0.141%, with a pH of 7.85, and electrical conductivity of 1.18 dSm^−1^. The soluble cations (mg per gram soil) were Ca^2+^ (380.0), Mg^2+^ (147.6), Na^1+^ (134.3), K^1+^ (19.5), N^3+^ (0.011), and P^3+^ (0.009). The soluble anions (mg per gram of soil) were: CO_3_^2−^ (9.0), HCO_3_^3−^ (6.6), Cl^1−^ (310.9), and SO_4_^2−^ (96.0). Plots (3 × 6 m^2^) were plowed and weeds were removed, before leveling and dissecting the soil. The space between lines was 10–15 cm, and the seeds were planted at a distance of 3–4 cm on the same line. Flooded irrigation was used. Plots were repeated in threes and organized in a randomized block design. The recommended growing practices of fenugreek plants were applied.

At the beginning of the flowering stage, twenty fenugreek plants were randomly selected and growth parameters (shoot length (cm), root length (cm), branches and leaves number per plant, and shoot fresh and dry weight per plant) were measured. During the harvest stage, yield parameters including, pods number per plant, the weight of 100 dry pods, the number of seeds per plant, and the total seed yield per hectare were recorded.

The disease development under field conditions was monitored by determining of incidence of *Rhizoctonia* root rot starting from 20 and up to 120 days from sowing for seed rot and plant mortality symptoms. 

### 2.10. Experimental Design and Statistical Analysis

Trials were accomplished in, at least, thrice, and the obtained data were subjected to statistical evaluation through the estimation of standard deviation (SD) for laboratory studies. The treatments of greenhouse and field studies were arranged in a randomized block design. After performing a one-way ANOVA, mean averages were compared based on the Tuckey test at probability ≤ 0.05. The software: CoStat (version 6.450, CoHort Software, Birmingham, UK) was used.

## 3. Results

### 3.1. Isolation and Antagonistic Activity of Endophytic Bacteria 

**Figure 1:**
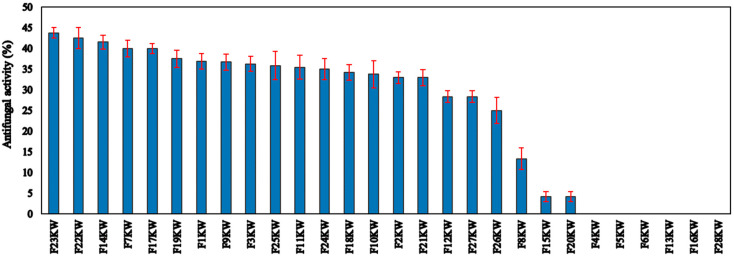
The antifungal activity of the isolated endophyte bacteria against *R. solani.* Red line is the standard deviation of the mean of the antifungal activity of the tested bacteria.

**Figure 2:**
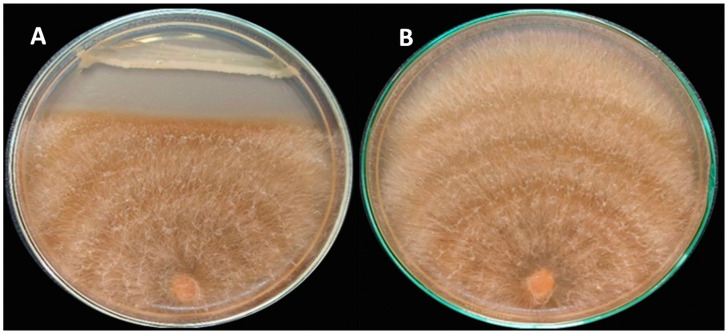
Plates of the in vitro anti-biological activity of the endophytic bacterium; F23KW against *R. solani* RT8, show the antagonism between the dual cultured *R. solani* and F23KW strain (**A**), in comparison with the solely grown *R. solani* on the PDA plate (**B**).

Pure cultures of endophytic bacteria were separated from fenugreek seeds. The isolates were screened regarding the activity against the causative pathogen of root rot disease (*R. solani*) of fenugreek plants. Data are depicted in Figure 1 in descending order. Most of the endophytic isolates showed obvious activity toward the fungal pathogen. The strain No. F23KW was the highest endophytic bacterium, causing the highest antagonistic activity against *R. solani*, reaching up to 43.75% Figure 2; however, 6 isolates were found to be sluggish in growth and inactive in biological activity. Of the isolates, 10 were selected for further biochemical tests.

### 3.2. Profile of Biochemical Features 

The pattern of lytic enzymes of the most 10 active bacterial isolates was explored to elucidate the hydrolysis capacity of each bacterium (Table 1). The pattern varied according to the enzyme, and the isolate. Most isolates were able to produce xylanase, FPase, PGase, and chitinase. On the other side, proteinase was secreted by a limited number of isolated bacteria (F22KW, F23KW, and F25KW). The isolates number F23KW and F25KW demonstrated a high ability to secrete all the tested enzymes compared to the other isolates. The isolated bacteria were found to produce moderate amounts of IAA. Isolate number F23KW was superior in this respect. Finally, the pH of the collected supernatants lay in a very narrow range; 8.2–8.5.

### 3.3. GC-MS Profile

**Figure 3:**
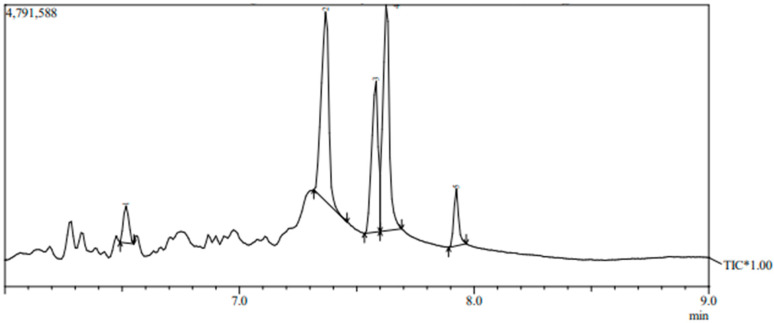
GC-MS chromatogram of bioactive metabolites of bacterial strain number F23KW. * = Total ion chromatogram of the tentatively identified compounds by GC-MS. Arrows represent the beginning and end of the base of the compound peak.

For further elucidating of the bioactive metabolites, the profile of the active compounds secreted in the growth medium by the selected bacterial strain No. F23KW was specified using GC-MS (Figure 3 and Table 2). Five dominant compounds were identified. The major compounds were methane, diethoxy-(37.20%), 2-Propanol, 1,1′-oxybis- (27.37%), and Propane, 1,2-dimethoxy-(22.66%). Additionally, two compounds were detected in minor amounts i.e., Dipropylene glycol monomethyl ether (6.43%), and 2-Butanol, 3,3’-oxybis-(6.35%).

### 3.4. Bacterial Identification

Based on the preceding data, the bacterium isolate number F23KW was selected for identification. The morphological and biochemical investigations (Table 3) revealed that the bacterium was Gram-negative straight rods that did not form spores and was strictly aerobic. The bacterium was motile with peritrichous flagella. The strain failed to utilize citrate, hydrolyze gelatin, or production of H_2_S; it produces IAA and can hydrolysis of lipids. The strain F23KW was positive for chitinase, xylanase, cellulase, pectinase, oxidase, catalase, nitrate reductase, indole test, and citrate utilization but negative for urease; it produced acid oxidatively from xylose, but not from lactose, maltose, mannitol, or sucrose. The isolate was identified as *Achromobacter* sp. strain F23KW.

**Figure 4:**
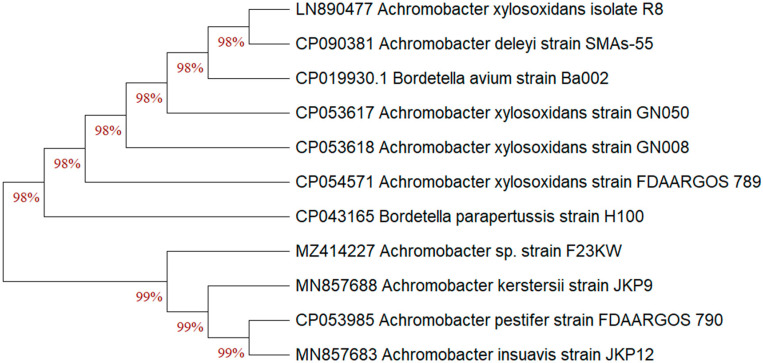
The neighbor-joining algorithm phylogenetic tree of *Achromobacter* sp. strain F23KW with respect to the closely related species of the genus on the Gene bank.

The previous identification was confirmed since the selected bacterial strain was molecularly characterized. After PCR amplification, the sequence that codes for the 16S rRNA gene were assessed. From the Blast assessment, the isolate F23KW exhibited a high resemblance with the earlier identified *Achromobacter* sp. on the GenBank (Figure 4). The percentage of replicate trees that clustered similar bacteria together in the bootstrap test is presented below the branches; this analysis involved 11 nucleotide sequences. Accordingly, the strain was identified as *Achromobacter* sp. strain F23KW. The GenBank accession number of the present current strain was MZ414227.

### 3.5. Greenhouse Evaluation

#### 3.5.1. *Achromobacter* sp. F23KW vis *R. solani*

Under infection stress, data organized in Table 4 show a significant (probability ≤ 0.05) reduction in the percentage of *Rhizoctonia* infection either on seeds or at the seedlings stage as a result of *Achromobacter* sp. F23KW application. in this respect, no significant variations were observed among all the bacterial treatments in reducing the seed rot and seedlings mortality in comparison to the untreated-infected control. Without infection stress, a positive impact was recorded among all the bacterial treatments in increasing percentages of fenugreek seedlings’ survival.

#### 3.5.2. Growth Features of Fenugreek Seedlings

The vegetative growth features of fenugreek seedlings as a reaction to different *Achromobacter* sp. F23KW treatments were followed up (Table 5). Under infection stress, the highest significant rise was recorded in shoot length due to BS and BCS, being 140.38 and 143.13%, respectively. BC and fungicide application came in the second increasing order, recording 89.56 and 66.21.7%, respectively, in comparison to pathogen only. Similar increments were recorded in root length and plant leaves number due to the treatment with BS, BC, or BCS (44.74, 39.47, 68.42%, and 76.19, 42.86, 66.67%, respectively) in comparison to infected plants. The positive influence of the tested bacterial treatments outspread to the fresh and dry weights, reaching 50.90 and 91.49%, 64.07 and 95.74%, and 62.28 and 121.27%, respectively, in the case of BC, BS, and BCS in relation to the infected control. Under the absence of the fungal pathogen, BCS showed the maximum significant increase in all fenugreek growth features as compared with the noninfected negative control. 

#### 3.5.3. Physiological Performance of Fenugreek Seedlings

The physiological recital of fenugreek seedlings was screened in terms of measuring the total phenols, PPO, and POD as defense-related enzymes (Table 6). In the presence of *R. solani*, a significant rise was detected in total phenol as a reaction to BCS application (482.17 mg/100 g fresh weight), compared to fungicide treatment and untreated-infected control (407.0 and 392.68 mg/100 g fresh weight, respectively). On the other side, the PPO activity was significantly increased due to BCS application (40.3 U), followed by BC, BS, and fungicide treatments, recording 28.0, 28.2, and 29.0 U, respectively, as compared to infected control (13.0 U). Under infection stress also, the POD activity was dramatically increased by a sole BC (1588.67 U), followed by BCS (936.67 U) as compared to infected control (17.33 U). Without infection stress, generally, the bacterial treatments induced total phenols content, as well as PPO, and POD activities.

### 3.6. Performance of Fenugreek under Field Conditions

#### 3.6.1. Growth and Yield

**Figure 5:**
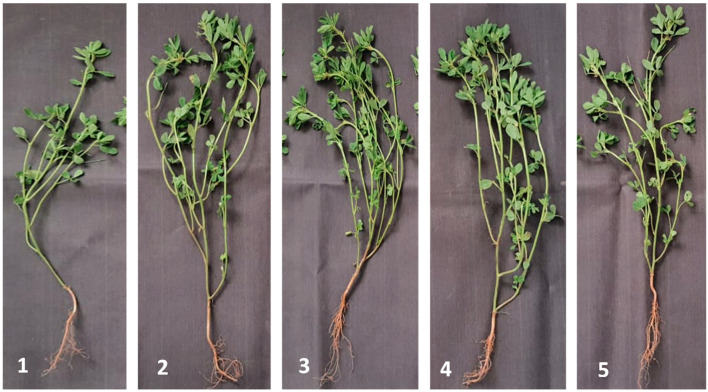
Fenugreek growth characteristics as affected by application of various endophyte *Achromobacter* sp. F23KW treatments under field conditions. The negative control without any treatment (**1**), the single application of endophytic bacterial cells (**2**), the single application of endophytic bacterial supernatant (**3**), the mixture of bacterial cells and supernatant (**4**), and the recommended fungicide (**5**).

The endophytic *Achromobacter* sp. F23KW was evaluated under natural infection in field conditions. Data (Table 7 and Figure 5) show significant variations in growth features on fenugreek plants as a response to *Achromobacter* sp. F23KW treatments. Seeds treated with BCS, BS, or BC exhibited the highest increase in shoot and root lengths (39.7, 29.64, and 24.62%, and 43.06, 37.5, and 25.69%, respectively) as compared to the control treatment. Similar increments were recorded in branches number due to the same treatments; however, BC and BCS treatments showed the maximum significant increase in leaves number (55.14 and 78.92%, respectively) as compared to control. No significant differences were observed among all biotreatments on plant fresh and dry weights and the chemical fungicide; however, treatment with BCS showed the highest significant rise in pods number per plant, the weight of 100 dry pods, number of seeds per plant, and total yield, being 125.23, 47.1, 71.69, and 60.88% increase over the control, respectively. The other two biotreatments (BS or BC) and fungicide came next as compared to the control.

#### 3.6.2. Disease Progress under Field Conditions

**Figure 6:**
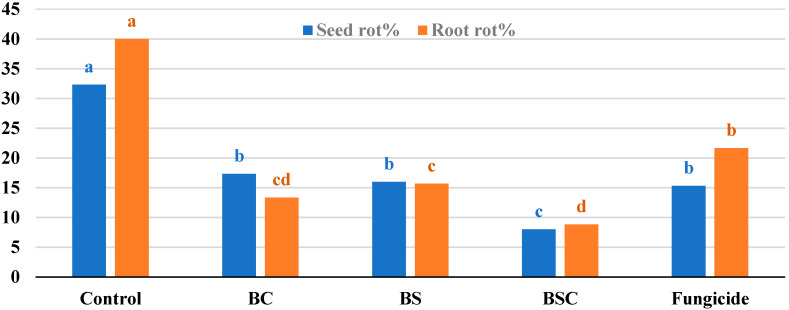
Seed rot and root rot (plant mortality) of fenugreek as a response to biotreatments of *Achromobacter* sp. F23KW under field conditions. Columns for each criterion superscripted by a different letter(s) are significantly different (Tukey test at probability ≤ 0.05).

Monitoring disease development under natural field infection (Figure 6) reveals that all biotreatments significantly lessened the incidence of disease symptoms on fenugreek during the growing season. The remedy of BSC was a highly efficient treatment in the reduction of the disease incidence of seed rot (8.0%) and plant mortality (8.83%) symptoms. Treated seeds with BC, BS, and the chemical fungicide ranked second in reducing the symptom of rotted seeds, while both biotreatments came third after fungicide in decreasing plant root rot symptoms as compared to control.

## 4. Discussion

*Rhizoctonia solani* is one of the widespread devasting fungal diseases in fenugreek-growing fields, causing root rot. Current antifungals are expensive, and their overuse is leading to the emergence of resistant strains and relapse of infections. Protection of plant seedlings from soil-borne and seed-borne pathogens through seed-coating with microbial inoculants is an eco-friendly way to manage root rot diseases in many crops [11].

In our isolation trials, several endophytic bacteria were isolated from fenugreek seeds. Over the last decades, a substantial amount of data confirmed the talented use of endophytic microbes to manage diseases. Endophytes include the cluster of microorganisms that inhabit intra-and/or intercellularly of the plants’ tissues without apparent signs on the host plants; these microorganisms are considered a potential resource of novel bioproducts for various agricultural, industrial, and medicinal aspects, such as anticancer, antibiotics, and biological control agents. Several antifungal mechanisms of action of the microbial endophytes were reported, such as the secretion of catalytic substances like chitinase and/or the production of antimicrobials [32,33]. In general, the spaces between cells in seeds are a suitable place for the living of several endophytic bacterial microbiomes, possibly due to the high content of the intercellular spaces with amino acids, carbohydrates, and inorganic nutrients [34].

The isolated endophytes were screened for their antagonistic capabilities to control *R. solani*, of them, 10 isolates showed a marked effect on the pathogen; however, there are several reasons for the biological activity of microorganisms. The enzymatic profile of endophytic bacterial microbiomes was explored as a suggested biological activity. In this connection, among the isolated endophytes *Achromobacter* strains from medicinal plants roots of *Ziziphora capitata*, and *Hypericum perforatum*; a strain of *A. piechaudii* S7 showed the maximum percentages growth inhibition of six fungal plant pathogens, including *Fusarium oxysporum* f. sp. *radicis-lycopersici*, *F. solani*, *F. culmorum*, *Gaeumannomyces graminis* pv. *tritici*, *Alternaria alternata*, *Botrytis cinerea*, and the oomycete *Pythium ultimum*. The antifungal action was ascribed to several enzymes e.g., proteases, cellulase, β-1,3-glucanase, and the production of IAA, and hydrogen cyanide (HCN) [32]. Recently, a strain of *A. xylosoxidans* CTA8689 was able to repress the *Fusarium solani* in dual culture (58.67%). The mechanical effect of the strain CTA8689 was proficiency to secrete volatile compounds that inhibit the growth of mycelia and spore germination of the pathogen, as well as its lytic enzymes like chitinase, protease, and pectinase [11].

Therefore, the lytic activity was explored during growth on fenugreek straw. The tissue structure of fenugreek plants is rigid to disintegrate because it is composed primarily of hemicellulose, cellulose, pectin, and protein. In general, such a structure restricts the activity of the endophytic microorganisms inside plant tissues. Therefore, for the selection of active endophytic bacterium, the various hydrolytic enzymes were assayed; these enzymes are reported to be included during the pathogenicity course, therefore, are regarded as cell wall-degrading enzymes, and can aid in the penetration of plant tissue by the fungal hyphae [12,13]. The great inconsistency in the lytic activity profile amongst bacterial isolates may be owed to the strain type, and the genetic disparity [10].

The overall cellulolytic activity of the isolated bacteria was measured as FPase. Cellulose is a polymer that is degraded by cellulases into single glucose units [10]. Similarly, xylanase manages the hydrolysis of hemicellulose (xylan), resulting in the liberation of xylose units [14,15]. Pectin is another complicated polymer, that contributes to the plant cell structure. PGase is the main pectin-catalyzing enzyme that cleaves the α-1,4-glycosidic bond of pectin into galacturonic acid [12,16]. Proteases hydrolyze the protein part of the plant tissues into amino acids, and peptides [13] through exopeptidases, and endopeptidases, that catalyze the terminal, and nonterminal peptide bonds, respectively [35]. The united action of such lytic enzymes led to the disintegration of plant tissue, which eases the microbial route into plants [13] and, therefore, may be vital for endogenous microorganisms.

The chitinolytic activity was noticed in limited numbers of the tested bacteria. The enzyme hydrolyzes the 1-4 β-glycoside linkage of N-acetyl-D-glucosamine in fungal chitin in the cell; thus, chitinase acts as a biofungicide and bioinsecticide component [17,18]. Chitinolytic microbes can utilize chitinase to antagonize the other pathogenic microorganisms that are present in host plants [13]. The current fermentation medium was free from the chitin, indicating that chitinase is biosynthesized constitutively by the positive bacteria. The secretion of the microbial enzymes may be through (1) stimulation by a specific substrate, (2) constitutive that does not need the existence of a substrate, or (3) by both mechanisms [12]. Collectively, *Achromobacter* sp. strain F23KW has a complete profile of hydrolytic enzymes in high amounts compared with the other isolates.

The IAA auxin is another promoter factor that was reported to be secreted by endophytic bacteria [11,33]. The current endophytes produced a moderate amount of IAA, which is known to be found in the new tissues, such as the young leaves, and apex to regulate cell division and elongation, leaf morphogenesis, and vasculature network developments, as well as regulate the primary and lateral root development and delineation, also regulate the responses to light, and gravity [19].

Among the endophytic isolates, an active bacterium, which exhibited high lytic activity and antagonistic capability against *R.* *solani,* was identified as *Achromobacter* sp. F23KW and the 16S rRNA sequencing was registered on the Gene bank under the accession number; MZ414227. Based on the morphological, biochemical, and molecular identification, the isolate was identified as *Achromobacter* sp. strain F23KW, which is included in the genus *Achromobacter*, under the family *Alcaligenaceae* in the order *Burkholderiales* [24]. Molecular identification is usually performed because of its sensitivity and specificity for the rapid identification of various organisms [36]. The sequence used for constructing the phylogeny is, totally, interpreted and firmly correlated with the other similar bacterial strains in the GeneBank database. The strain was identified as *Achromobacter* sp. strain F23KW, which came in line with previous identification tests.

The GC-MS profile of the selected; *Achromobacter* sp. F23KW showed the occurrence of highly concentrated substrates. Methane, diethoxy- was the highest component in the bacterial supernatant, also has several names as, diethoxymethane, glyme, monoglyme, dimethyl glycol, and ethylene glycol dimethyl ether, the compound is a colorless volatile liquid with an agreeable odor, less dense than water, and the vapors are heavier than air. Maybe narcotic in high concentrations. Used as a solvent, especially in batteries and in the manufacture of cosmetics; it is miscible with water [37]. In the recent pharmaceutical industry, diethoxymethane has been recommended as a renewable solvent for Pd-catalyzed carbonylations [38]; these features and uses suggest the compound is a promising antimicrobial.

The second major component was 2-Propanol, 1,1′-oxybis-, it is a nearly odorless, colorless, and slightly viscous liquid; this compound is approved as a pesticide in the USA; it is also used as a solvent in the production of polyester and alkyd resins, reinforced plastics, and plasticizers; it is included in the printing industry, in antifreeze, and in air sanitation, since its vapor is highly germicidal; this volatile compound was reported in lemon balm essential oils, which have fungistatic and fungicidal effects against the growth of ochratoxigenic *Penicillium verrucosum* [39], and mycotoxinogenic *Fusarium culmorum* and *F. proliferatum* [40]. Additionally, 2-Propanol, 1,1′-oxybis- was detected in the chemical composition of rosemary essential oil, which has insecticidal action versus *Galleria mellonella* (greater wax moth) [41].

The third major compound, propane, 1,2-dimethoxy-, is a colorless liquid used in water-sensitive reactions as a water scavenger, this feature can be used to accurately determine the amount of water in a sample [42]. There are no reports on the biological activity of this compound.

Dipropylene glycol monomethyl ether is an organic solvent with a wide variety of commercial and industrial applications [43]. Like other glycol ethers, it is used as a solvent in printing ink paints and coatings, as well as an anti-freezing in diesel engines [44]. An in vitro study reported propylene glycol as an antimicrobial versus *Candida albicans*, *Staphylococcus aureus*, *St. epidermidis*, *St. pyogenes* A, *Streptococcus mitis*, and *Escherichia* [45]. Additionally, the bactericidal effect of propylene glycol was previously emphasized versus *Enterococcus faecalis, Escherichia coli*, and *Streptococcus mutans* [46].

Finally, 2-Butanol, 3,3’-oxybis- was identified among the bacterial supernatant, which was reported as a prevalent volatile organic compound emission from all regular essential oils such as orange, coriander, thyme, and jasmine essential oils, which have strong antimicrobial activity against several bacterial pathogens [47,48,49].

Despite the GC-MS of the supernatant of the current bacterial strain showing little diversity in the metabolites, they are secreted in highly concentrated amounts, indicating the relative homogeneity of the metabolites. Furthermore, the majority of the detected components were reported to have antimicrobial activity.

In the in vivo studies, *Achromobacter* sp. F23KW exerted optimistic action on plant growth and physiological performance, and further managed the root rot disease of fenugreek plants. Similarly, the endophytic bacterium *A. xylosoxidans* has proven its ability to promote plant growth and control *F. oxysporum* in tomatoes, and radish [50,51]. Under a greenhouse experiment, rice plants treated by *A. xylosoxidans* under infection with *Magnaporthe oryzae* showed a significant decrease in blast disease incidence by 39%, and a rise in the performance of defense-related enzymes like POD, PPO, and phenyl ammonia lyase, as well as chitinase, besides improving the plant height, and yield [52].

Several beneficial roles of endophytic bacteria on the host plant were reported, where they can promote growth, modulate the metabolism, and phytohormone that enhances plant resistance toward various pathogens and environmental stresses [53]; moreover, inoculation of rice plants by the endophytic *A. xylosoxidans* WM234C-3 led to a marked increase in the root, and shoot lengths, fresh weight, and chlorophyll [54]. Another, the endophytic *Achromobacter xylosoxidans* AUM54 can enzymatically reduce deleterious ethylene levels and increase the activity of antioxidant enzymes like catalase, ascorbate-peroxidase, and superoxide dismutase in *Catharanthus roseus* [55]. One more role was reported by *A. xylosoxidans* strain Ax 10 that enables *Brassica* sp. plants to mitigate stress when grown in copper-contaminated soils [56]. The significant improvement of vegetative growth parameters by the endophytic *A. xylosoxidans* may be attributed to its growth-promoting activities, including IAA and gibberellin secretion, fixation of nitrogen, and solubilizing of complex phosphate, as well as the heightening of photosynthetic pigments and N, P, and K absorption [11,57].

Under field evaluation, the current study reported obvious improvements in the performance of fenugreek growth, yield, and disease management. In this connection, singularly or combined bio inoculation by endophytic *Azospirillum brasilense* and/or *A. lipoferum* contributed remarkably to maize and wheat yields [58]; moreover, the inoculation by a consortium of deaminase-active endophytic bacteria has a significant influence on growth parameters especially plant height, productive tillers, panicle and spike lengths, harvest index, and grain, and straw yields, as well as the nutrient contents of rice and wheat [59]; however, several means were reported to promote plant growth, and physiology by endophytic bacteria, including increasing nutrient availability through phosphorus solubilization, and nitrogen fixation, siderophore production, modulating the stress responses of the plant via phytohormone biosynthesis, and finally, bio controlling pathogens via the secretion of antimicrobials [60].

Under natural conditions, epiphytic bacteria can inhabit seeds and systemically colonize the plants during the growth cycle, leading therefore to the management of soil-borne pathogens [60]. For instance, *A. xylosoxidans* significantly suppressed the leaf blight caused by *Xanthomonas oryzae* pv. *oryze* [61]. Similarly, under field trial, the combined inoculum with a consortium composed of the endophytic bacteria (*A. piechaudii* AP_RD9, *Pseudomonas* sp. PCh_RH1, and PG_RD39, and *Stenotrophomonas* sp. SS_RD24) under nutrient limiting conditions increased the yield of oilseed by 15% and reduced the severity of *Phoma* stem canker disease by 42% [60].

The protective role of the endophytes may be owing to the stimulation of resistance by jasmonic, and salicylic acids signaling and/or the production of the antimicrobial substances [62]. The mechanisms of protection also include the increment of lytic enzymes, expression of pathogenesis-related proteins, and synthesis of antifungal substances [63,64]. Our investigation described the secretion of various hydrolytic enzymes; moreover, our GC-MS showed the occurrence of components with antimicrobial action.

## 5. Conclusions

Summing up, to our knowledge, this is a novel endophytic bacterial isolate applied as a natural bioproduct for used dually as a growth promoter and biocontrol agent against the sclerotia-forming; *R. solani*, (the causative agent of fenugreek root rot); this novel bacterial strain has proven its ability to manage growth and root rot disease under various in vitro and in vivo circumstances; however, additional investigation is encouraged to evaluate the current endophytic bacterium on other plants and under various environmental setups.

## Figures and Tables

**Table 1:** Enzymatic profile, indole acetic acid biosynthesis, and final pH of the endophytic bacterial isolates.

Isolate	Xylanase (U)	FPase (U)	PGase (U)	Proteinase (U)	Chitinase (U)	IAA (µg/g)	Final pH
F1KW	61.92 ± 5.01	3.20 ± 0.97	2.91 ± 0.27	ND	0.19 ± 0.04	242.8 ± 4.8	8.4 ± 0.5
F3KW	60.61 ± 4.12	3.14 ± 0.55	1.49 ± 0.64	ND	0.57 ± 0.12	255.1 ± 9.5	8.4 ± 0.3
F9KW	55.78 ± 3.41	2.89 ± 0.61	2.53 ± 0.81	ND	1.10 ± 0.12	119.3 ± 8.2	8.4 ± 0.4
F7KW	67.63 ± 3.57	3.50 ± 0.45	2.34 ± 0.93	ND	2.30 ± 0.91	205.8 ± 5.9	8.3 ± 0.2
F14KW	65.44 ± 2.56	3.39 ± 1.01	2.30 + 0.34	ND	0.53 ± 0.27	242.8 ± 4.9	8.2 ± 0.6
F17KW	ND	ND	2.03 ± 0.54	ND	ND	144.0 ± 7.3	8.2 ± 0.2
F22KW	59.73 ± 3.39	3.09 ± 0.99	1.76 ± 0.09	8.47 ± 1.33	ND	170.0 ± 6.1	8.3 ± 0.3
F23KW	70.71 ± 4.47	3.50 ± 0.83	3.10 ± 1.00	7.50 ± 1.96	6.20 ± 1.29	353.9 ± 5.6	8.5 ± 0.4
F19KW	65.44 ± 1.95	3.39 ± 0.92	1.53 ± 0.82	ND	1.10 ± 0.99	218.1 ± 5.8	8.5 ± 0.1
F25KW	67.63 ± 4.85	3.66 ± 0.31	1.72 ± 0.39	7.23 ± 2.47	4.80 ± 1.38	157.6 ± 3.9	8.4 ± 0.5

ND: no activity was detected.

**Table 2:** Quantitative of the detected compounds in the hydrolysate of the supernatant of the bacterial strain number F23KW using GC-MS.

Peak No.	Retention Time (min)	Compound	Formula	Molecular Weight	Retention Index	Area	Height	Area Sum, %
1	6.516	Dipropylene glycol monomethyl ether	C_7_H_16_O_3_	148.200	1034	562,758	269,890	6.43
2	7.386	2-Propanol, 1,1’-oxybis-	C_6_H_14_O_3_	134.174	1018	2,395,965	793,699	27.37
3	7.580	Propane, 1,2-dimethoxy-	C_5_H_12_O_2_	104.148	859	1,984,213	979,929	22.66
4	7.627	Methane, diethoxy-	C_5_H_12_O_2_	104.148	843	3,256,992	1,456,742	37.20
5	7.924	2-Butanol, 3,3′-oxybis-	C_8_H_18_O_3_	162.227	1089	555,580	339,089	6.35

**Table 3:** Morphological and biochemical features of the selected endophytic bacterial strain F23KW.

Taxonomic Feature	Strain F23KW
General features	
Gram reaction	−
H_2_S production	−
Lipid hydrolysis	+
Gelatin hydrolysis	−
Citrate utilization	−
Phenylalanine deamination	−
Indole test	+
Indole-3-acetic acid	+
Carbohydrate fermentation	
Xylose	+
Maltose	+
Lactose	−
Maltose	+
Mannitol	−
Sucrose	+
Mannose	−
Fructose	+
Tolerance to NaCl (% *w*/*v*)	Up to 3.0
pH range of growth	6–9.0
Enzymatic activity	
Chitinase	+
Xylanase	+
Cellulase	+
Pectinase	+
Proteinase	+
Urease	−
Oxidase	+
Catalase	+
Nitrate reductase	+

+ Positive result, − negative result.

**Table 4:** Effect of *Achromobacter* sp. F23KW on *Rhizoctonia* root rot development on fenugreek seedlings under greenhouse situations.

Treatment		Pre-Emergence Damping Off, % *	Post-Emergence Damping Off, % *	Plant Survival, % *
Infected	P	25.0 a	17.2 a	57.8 c
	PBC	14.2 b	10.2 b	75.6 b
	PBS	13.2 b	10.8 b	76.0 b
	PBCS	11.8 b	8.20 bc	80.0 b
	PF	13.2 b	10.2 b	76.6 b
Noninfected	Control	3.40 c	3.20 c	93.4 a
	BC	5.60 c	5.00 bc	89.4 a
	BS	5.80 c	4.00 c	90.2 a
	BCS	5.40 c	3.80 c	90.8 a

* Pre-emergence damping off is the percentage of seed and seedling death before germination after 2 weeks. Post-emergence damping off is the percentage of seedling death after germination, and survival is the percentage of living plants at the end of the 60 days growth period. P; *R. solani* pathogen only, PBC; P plus endophytic bacterial cells, PBS; P plus endophytic bacterial supernatant (PBS), PBCS; P plus a mixture of PBC and PBS, PF; P plus the recommended fungicide, Control; the negative control without any treatment, BC; the single application of endophytic bacterial cells without infection, BS; the single application of endophytic bacterial supernatant without infection, and BCS the mixture of BC and BS without infection. Means are the average of 15 replicates (pots). Within each column, a different letter(s) indicates significantly different (Tukey test at probability ≤ 0.05).

**Table 5:** Fenugreek growth as influenced by endophyte Achromobacter sp. F23KW treatments under greenhouse conditions.

Treatment		Plant Length (cm)		Leaves Number per Plant	Plant Weight (g)	
		Shoot	Root		Fresh	Dry
Infected	P	7.3 c	3.8 c	4.2 c	334.0 c	47.0 d
	PBC	13.8 b	5.5 ab	7.4 a	504.0 ab	90.0 ab
	PBS	17.5 a	5.3 ab	6.0 ab	548.0 ab	92.0 ab
	PBCS	17.7 a	6.4 a	7.0 ab	542.0 ab	104.0 ab
	PF	12.1 b	4.9 bc	5.6 bc	412.0 bc	61.8 cd
Noninfected	Control	14.0 b	4.8 bc	6.2 ab	495.8 ab	83.8 bc
	BC	14.8 ab	6.0 ab	7.2 ab	516.0 ab	90.6 ab
	BS	17.3 a	6.2 ab	7.0 ab	552.0 ab	93.0 ab
	BCS	17.8 a	6.4 a	7.6 a	576.0 a	112.6 a

Means followed by a different letter(s) within each column are significantly different (Tukey test at probability ≤ 0.05). P; *R. solani* pathogen only, PBC; P plus endophytic bacterial cells, PBS; P plus endophytic bacterial supernatant (PBS), PBCS; P plus a mixture of PBC and PBS, PF; P plus the recommended fungicide, Control; the negative control without any treatment, BC; the single application of endophytic bacterial cells without infection, BS; the single application of endophytic bacterial supernatant without infection, and BCS the mixture of BC and BS without infection.

**Table 6:** Physiological characteristics of fenugreek as affected by endophyte *Achromobacter* sp. F23KW treatments under greenhouse conditions and *Rhizoctonia solani* infection.

Treatment		Total Phenols (100 mg/g)	Polyphenol Oxidase (U)	Peroxidase (U)
Infected	P	392.68 d	13.0 c	17.33 d
	PBC	405.02 cd	28.0 b	1588.67 a
	PBS	435.09 bcd	28.2 b	566.67 cd
	PBCS	482.17 b	40.3 a	936.67 bc
	PF	407.03 cd	29.0 b	513.34 cd
Noninfected	Control	459.13 bc	23.0 bc	788.67 bc
	BC	440.09 bcd	29.0 b	1045.33 abc
	BS	484.17 b	42.0 a	1361.33 ab
	BCS	565.32 a	49.0 a	766.00 bc

Means followed by a different letter(s) within each column are significantly different (Tukey test at probability ≤ 0.05). P; *R. solani* pathogen only, PBC; P plus endophytic bacterial cells, PBS; P plus endophytic bacterial supernatant (PBS), PBCS; P plus a mixture of PBC and PBS, PF; P plus the recommended fungicide, Control; the negative control without any treatment, BC; the single application of endophytic bacterial cells without infection, BS; the single application of endophytic bacterial supernatant without infection, and BCS the mixture of BC and BS without infection.

**Table 7:** Growth and yield of fenugreek as affected by the tested endophyte *Achromobacter* sp. F23KW treatments in the field under natural infection conditions.

Treatment	Shoot Length (cm)	Root Length (cm)	Branches Number/Plant	Leaves Number/Plant	Plant Fresh Weight (g)	Plant Dry Weight (g)	Pods Number/Plant	Weight of 100 Dry Pods	No. Seeds/Plant	Total Yield (ton/h)
Control	39.8 b	14.4 d	3.6 c	37.0 b	17.39 c	3.01 b	11.1 b	26.20 d	294.33 e	0.294 e
BC	49.6 a	18.1 bc	5.8 ab	57.4 a	29.37 ab	4.11 ab	18.8 ab	33.52 b	408.67 c	0.414 c
BS	51.6 a	19.8 ab	5.6 ab	49.4 ab	31.96 ab	4.46 a	20.0 ab	33.48 b	440.00 b	0.438 b
BCS	55.6 a	20.6 a	6.4 a	66.2 a	36.16 a	4.77 a	25.0 a	38.54 a	505.33 a	0.473 a
Fungicide	48.0 ab	16.6 c	4.8 bc	50.6 ab	27.01 b	3.97 ab	18.0 ab	29.90 c	371.67 d	0.359 d

Means followed by a different letter(s) within each column are significantly different (Tukey test at probability ≤ 0.05). Control; the negative control without any treatment, BC; the single application of endophytic bacterial cells, BS; the single application of endophytic bacterial supernatant, BCS; the mixture of BC and BS.

## Data Availability

The relevant data applicable to this research are within the paper.

## Sample Availability

Samples of the compounds are not available from the authors.
